# Prevention of Teratogenesis in Pregnancies of Obese Rats by Vitamin E Supplementation

**DOI:** 10.3390/antiox10081173

**Published:** 2021-07-23

**Authors:** Martin Alcala, Victoria E. Bolado, Isabel Sánchez-Vera, Sonia Clapés, Francisco Dasí, Guillermo Sáez, Esther Carrera, Fabiola Alvarez-Gallego, Mary R. Loeken, Marta Viana

**Affiliations:** 1 Departamento de Química y Bioquímica, Facultad de Farmacia, Universidad CEU-San Pablo, CEU Universities, 28668 Madrid, Spain; f.alvarez17@usp.ceu.es; 2 Laboratorio de Investigación Genómica y Fisiológica, Facultad de Nutrición, Universidad Veracruzana, Xalapa 91090, Mexico; vicboga@hotmail.com; 3 Departamento de Ciencias Médicas Básicas, Facultad de Medicina, Universidad CEU-San Pablo, CEU Universities, 28668 Madrid, Spain; isanver@ceu.es; 4 Departamento de Bioquímica del Instituto de Ciencias Básica y Preclínica “Victoria de Girón”, Universidad de Ciencias Médicas de la Habana, La Habana 10699, Cuba; sclapes@infomed.sld.cu; 5 Department of Physiology, Instituto de Investigación Sanitaria INCLIVA, University of Valencia, 46010 Valencia, Spain; francisco.dasi@uv.es; 6 Departamento de Bioquímica y Biología Molecular, Facultad de Medicina y Odontología-INCLIVA, Servicio de Análisis Clínicos, Hospital Universitario Dr.Peset-FISABIO, Universitat de Valencia, 46017 Valencia, Spain; guillermo.saez@uv.es; 7 Departamento de Ciencias Farmacéuticas y de la Salud, Facultad de Farmacia, Universidad CEU-San Pablo, CEU Universities, 28668 Madrid, Spain; escarrera@ceu.es; 8 Section on Islet Cell and Regenerative Biology, Joslin Diabetes Center, Department of Medicine, Harvard Medical School, Boston, MA 02215, USA; mary.loeken@joslin.harvard.edu

**Keywords:** embryo malformation, teratogenesis, oxidative stress, glutathione, vitamin E, obesity

## Abstract

Congenital malformations are a common adverse outcome in pregnancies complicated by pregestational obesity, although the underlying mechanisms are still unrevealed. Our aim was to study the effect of oxidative stress in obesity-induced teratogenesis. Wistar rats were fed a high-fat diet for 13 weeks, with (OE group) or without (O group) vitamin E supplementation. Then, rats were mated and sacrificed at day 11.5 of gestation. Embryos from O dams presented a 25.9 ± 3.5% rate of malformations (vs. 8.7 ± 3.4% in C rats), which was reduced in the OE group (11.5 ± 2.3%). Pregestational obesity induced hepatic protein and DNA oxidation and a decline in antioxidant enzymes. Importantly, glutathione content was also decreased, limiting the availability of this antioxidant in the embryos. Vitamin E supplementation efficiently maintained glutathione levels in the obese mothers, which could be used in their embryos to prevent oxidation-induced malformations. To test the effect of decreasing glutathione levels alone in a cell culture model of neuroepithelium, murine embryonic stem cells (ESC) were induced to form neuronal precursors and glutathione synthesis was inhibited with the gamma–glutamylcysteine synthesis inhibitor, buthionine sulfoximine (BSO). BSO inhibited the expression of *Pax3*, a gene required for neural tube closure that is also inhibited by oxidative stress. Taken together, our data indicate that obesity causes malformations through the depletion of maternal glutathione, thereby decreasing glutathione-dependent free radical scavenging in embryos, which can be prevented by vitamin E supplementation.

## 1. Introduction

The prevalence of obesity is experiencing an alarming increase worldwide. A special focus should be the increase of obesity rates among women of reproductive age [1]. According to the National Health and Nutrition Examination Survey (NHANES), obesity prevalence (Body Mass Index ≥ 30 kg/m^2^) in women between 20 and 39 years of age reached 37% in 2013–2014 in the United States [2], and it is estimated that half of women attending the first antenatal visit are overweight. In Europe, the estimates of obesity prevalence vary from 7.1% in Poland to 25.2% in the United Kingdom [3]. Different meta-studies have reported an average global prevalence of obesity during gestation ranging between 1.8% and 70.3% [4,5].

The importance of these numbers lies in the fact that pregestational obesity has been linked to an increased risk of adverse outcomes at every stage of pregnancy, including periconceptional difficulties and health consequences in the newborn [3,4,5,6,7]. During gestation, obese mothers have a higher risk of spontaneous and recurrent pregnancy loss [8], preeclampsia and gestational diabetes mellitus [9]. At delivery, the chances of requiring a cesarean intervention in obese mothers is double that of lean mothers, and the frequency of anesthetic complications or massive blood loss occurs in one of every three deliveries [4]. For the newborn, there is a higher risk of macrosomia and shoulder dystocia, as well as obesity later in childhood [10,11]. In addition, the rate of congenital malformations is increased, mainly due to neural tube and cardiac defects [12,13,14].

Congenital anomalies are the result of abnormal organogenesis in utero during the first trimester of gestation in humans. The mechanisms involved in obesity-mediated teratogenesis are still unrevealed. The fuel-mediated teratogenesis concept in diabetic pregnancies proposed that embryo malformation is due to over delivery of glucose and ketone bodies to the embryo, resulting in inappropriate organ development [15,16,17]. However, obese pregnancies are not necessarily hyperglycemic, and even if they are, whether teratogenesis is, in part, a result of other metabolic disturbances secondary to poor glycemic control is yet to be determined.

Oxidative stress (OS) has been proposed as a common underlying mechanism for malformations induced by either maternal diabetes or obesity [7,18,19]. Both obesity and pregnancy are, *per se*, situations characterized by an imbalance between the production of reactive oxygen species and their elimination by antioxidant defenses. In fact, increased markers of oxidative stress in pregnant women with pregestational obesity, in comparison to lean pregnant women, have been reported, which positively correlates with oxidative markers in the newborn [20,21]. OS has been suggested as a potential mechanism in the teratogenesis induced by ionizing radiation, cocaine and alcohol abuse, hypoxia, cigarette smoking, or drugs such as valproate and thalidomide, as well as by diabetes [18,22,23,24]. In addition to a potential direct effect on DNA damage and repair [22], OS inhibits the expression of *Pax3*, a gene that is expressed in neuroepithelium and neural crest cells and is required for neural tube closure and for cardiac outflow tract septation [19]. As a result of impaired *Pax3* expression, neuroepithelium and cardiac neural crest cells undergo apoptosis by a process that is dependent on the p53 tumor suppressor protein, leading to neural tube and cardiac outflow tract defects [25,26,27]. OS also inhibits the expression of *Pax3* in a murine embryonic stem cell (ESC) model of differentiating neuroepithelium [28], and the inhibition of *Pax3* expression leads to the activation of p53-dependent apoptotic pathways [29]. Thus, OS can be teratogenic by impairing the expression of genes that are required to prevent apoptosis during the development of embryonic structures.

Vitamin E, comprising eight vitamers (four tocopherols and four tocotrienols), is the most abundant liposoluble antioxidant compound in the human body. Among them, α-tocopherol accounts for 90% of vitamin E activity in human tissues [30]. During pregnancy, the recommended intake is 15 mg/day, which can be easily covered with a regular diet. However, the beneficial effects of additional supplementation is controversial [31,32]. However, there are insufficient data regarding the supplementation of vitamin E alone in mothers with pregestational obesity [7]. We have shown that vitamin E improves the metabolic, inflammatory and oxidative profiles in a mouse model of diet-induced obesity [33]. Furthermore, we showed that vitamin E supplementation reduces diabetes-induced teratogenesis [18]. Thus, we hypothesize that vitamin E supplementation may prevent the teratogenic effects of obesity by maintaining a proper oxidative balance. We tested this hypothesis using a rat model of high fat diet-induced obesity with or without vitamin E treatment, and with neuronal precursors derived from ESC with and without the induction of OS.

## 2. Materials and Methods

### 2.1. Animals and Diet

Female Wistar rats were purchased from Harlan Laboratories (UK). After 2 weeks of acclimatization, rats were randomized into 3 groups. The control group (C, *n* = 10) received a standard chow diet that provides 11% calories from fat (Harlan Teklad Global Rodent Diet 2014, Envigo, IN, USA). The obese group (O, *n* = 12) received a cafeteria diet (HF) that provides 29% calories from fat, adapted from Holemans, et al. [34]. The composition of the cafeteria diet is summarized in the Supplementary Table S1. The vitamin E-supplemented group (OE, *n* = 12) was fed the same cafeteria diet and received 150 mg of vitamin E dissolved in 200 µL of sunflower oil (DL-α Tocopherol acetate; Sigma-Aldrich, St. Louis, MO, USA) twice a week by oral gavage [18]. The dietary treatments and the vitamin E supplementation lasted 13 weeks. Food intake and the weight of the animals were recorded weekly. All animals had free access to food and water. Animal procedures were performed in accordance with the USP-CEU Ethical Committee for Animal Research.

Once a statistically significant difference in weight was observed between the control group and the two groups receiving the cafeteria diet, rats were mated with male rats that had been fed the standard chow diet. Gestational day 0.5 was defined as noon following the morning of a positive vaginal swab for the presence of spermatozoa. During gestation, every group was maintained under the same diet and the vitamin E supplementation was delivered daily. Rats were sacrificed at day 11.5 of gestation, corresponding to the end of the embryonic stage.

The maternal liver was immediately dissected and snap frozen in liquid nitrogen and stored at −80 °C. Blood was collected using tubes containing Na_2_EDTA, and plasma was separated from whole blood by centrifugation and was stored at −20 °C until further analysis.

### 2.2. Analysis of Embryo Morphology

Embryos were accessed by terminal cesarean section on day 11.5 of gestation. Uterine horns were dissected, and the embryos were collected within the intact amniotic sac and placed in saline solution. Embryos were carefully dissected free of extra-embryonic tissues under a binocular dissection microscope. Embryonic resorption was defined as the presence of a chorion without embryo or the presence of embryonic tissue lacking any identifiable morphology. Unresorbed embryos were visually analyzed for the presence of malformations by a single researcher for consistency. Normal embryos exhibited developmentally appropriate axial curvature, intact prosencephalon, mesencephalon and rhombencephalon, total closure of the neural tube, and normally placed and sized cardiac and otic vesicles and branchial arches. Embryonic malformation was designated as a significant deviation from normal morphology. Crown-rump distance and the number of somites were also measured as indexes of embryo size and development.

### 2.3. Metabolic Parameters

Glucose, triglycerides and non-esterified free fatty acids were assayed by enzymatic colorimetric tests (GOD-PAP, LPL/GOP-Trinder, Roche Diagnostics, Basil, Switzerland and HR Series NEFA-HR, Wako Diagnostics, Neuss, Germany). Plasma levels of insulin were measured using a Milliplex MADPK-71K adipokine kit (Merck-Millipore, Burlington, MA, USA) according to the manufacturer’s instructions. For estimation of insulin resistance, HOMA index was calculated as HOMA-IR = (FPG × FPI)/2430, where FPI was in microunits per milliliter and FPG in milligram per deciliter, as previously described [35].

### 2.4. Vitamin E Determination

α-tocopherol was detected by an HPLC using chromatographic methods as routinely employed by our laboratory [36,37]. Briefly, vitamin E was extracted from 200 μL samples of plasma with hexane, evaporated, and resuspended in 200 μL of methanol. To extract vitamin E from liver samples, 50 mg of tissue was homogenized in 100 µL of a 5 mM phosphate buffer, pH = 7.4, 1 mL of acetone and 50 µL of phenyldodecane as an internal standard. After sonication and centrifugation, the supernatant was encapsulated into an HPLC vial. A Nucleosil C-18 column (5 µm, 15 × 46 mm) placed in an oven at a constant temperature of 40 °C was used for the separation. A mixture of 95:5 methanol/water was used as a mobile phase at a constant flow rate of 2 mL/min. The chromatograph system was a Beckman Model 126 coupled to a UV detector (Beckman Model 168, Fullerton, CA, USA) in line with a fluorescence detector (Waters 474, Milford, MA, USA). All the solvents used were high purity for chromatography purchased from Scharlau (Barcelona, Spain).

### 2.5. Sample Preparation for Oxidative Stress Analysis

Liver samples were homogenized in a buffer containing 50 mM Tris, pH = 7.4 and 5 mM EDTA. To avoid oxidation of the samples in those aliquots intended to be used for markers of oxidative damage, 5 mM BHT was added. Tissues were disrupted using a TissueLyser (Qiagen, Hilden, Germany) after 3 cycles at 50 Hz and the resulting lysates were stored at −80 °C until analysis of antioxidant enzymes activities and oxidative damage markers.

### 2.6. Lipid Peroxidation Products (LPO)

Lipid peroxidation was determined using a commercial kit (Bioxytech LPO-586) from OxisResearch (Portland, OR, USA). The LPO-586 assay is based on the reaction of a chromogenic reagent, *N*-methyl-2-phenylindole (R1), with MDA and 4-hydroxyalkenals at 45 °C. One molecule of either MDA or 4-hydroxyalkenal reacts with 2 molecules of reagent R1 to yield a stable chromophore with maximal absorbance at 586 nm. Lipoperoxides concentration was expressed as nmoles/mg tissue.

### 2.7. Assay of Advanced Oxidation Protein Products (AOPP)

Hepatic AOPP were determined according to the method of Witko–Sarsat [38] with modifications. Briefly, under acidic conditions, AOPP promote the transformation of iodide to diatomic iodine, and this reaction can be followed spectrophotometrically at 340 nm (Beckman DU-640 spectrophotometer, Fullerton, CA, USA). Samples were prepared as follows: 50 µL of sample, 50 µL of 1.16 M potassium iodide and 100 µL of acetic acid were mixed in 950 µL of 10 mM, pH = 7.4 phosphate buffer. A calibration curve was prepared under the same conditions using chloramine-T (Sigma-Aldrich, St. Louis, MO, USA) as the standard. AOPP concentration was expressed as micromoles of chloramine-T equivalents per mg of analyzed tissue.

### 2.8. 8-Oxo-7,8-dihydro-2′-deoxyguanosine

8-oxo-7,8-dihydro-2′-deoxyguanosine (8-OHdG) was detected as previously described [39]. Briefly, hepatic DNA was extracted, purified and digested with DNase. A 5-μm Spherisorb ODS2 column (4.6 mm × 250 mm) controlled by a Waters 515 HPLC pump with a flow rate of 1 mL/min was used to separate 8-oxo-7,8-dihydro-2′-deoxyguanosine. The mobile phase used was 50 mmol/L potassium phosphate (pH 5.1) in 5% acetonitrile, and the retention time was 7.5 min. Electrochemical detection was performed using an ESA Coulochem II detector. Results were expressed as 8-OHdG/10^6^ dG.

### 2.9. Glutathione Detection

Total glutathione (GSH + GSSH) was determined using the protocol described by Tipple and Rogers [40]. Briefly, GSH was oxidized by 5,5′-dithiobis-(2-nitrobenzoic acid) (DTNB) resulting in the formation of GSSG and 5-thio-2-nitrobenzoic acid (TNB). Total GSSG was then reduced to GSH by glutathione reductase. The rate of TNB formation is proportional to the sum of GSH and GSSG present in the sample and was determined by measuring the formation of TNB at 412 nm. Results were expressed as nmoles/mg protein.

### 2.10. Antioxidant Enzymes Activity

Catalase (CAT) activity in 100 µL of the sample was measured by monitoring the disappearance of 100 µL of 30 mM hydrogen peroxide at 240 nm with time [41] a in a final volume of 1 mL with 50 mM phosphate buffer, pH = 7.

Superoxide dismutase (SOD) activity was measured by the inhibition of cytochrome C oxidation by a superoxide generation system with xanthine and xanthine oxidase. Briefly, 50 µL of the sample were combined in a spectrophotometric cuvette with 1 mL of 50 mM phosphate buffer at 37 °C, pH = 7.8, 50 µL of 0.1 mM cytochrome c and 250 µL of 0.5 mM xanthine. The reaction was initiated by the addition of 50 µL of 0.02 U/mg protein of xanthine oxidase and was measured at 546 nm with time.

Glutathione peroxidase (GPx) activity was measured by the oxidation of glutathione by GPx [42]. In a quartz cuvette, the following components were combined: 800 µL of 50 mM phosphate buffer, pH = 7.4 at 37 °C, 20 µL of 0.1 M reduced glutathione, 20 µL of 20 U/mL glutathione reductase and 50 µL of sample. The reaction was initiated by the addition of 20 µL of 30 mM t-butyl hydroperoxide. Oxidized glutathione is regenerated by glutathione reductase using NADPH + H+ as a cofactor. The reaction rate was measured by following the disappearance of NADPH + H+ at 340 nm.

Glutathione reductase (GR) activity was measured by monitoring the disappearance of NADPH + H+ at 340 nm after the regeneration of oxidized glutathione as described by Goldberg and Spooner [43].

All enzyme activities were determined using liver lysates and the specific activities were calculated as U/mg protein.

### 2.11. Hepatic and Embryo Gene Expression Analyses

Liver gene expression analyses were performed using RNA extracted from five independent samples from each experimental treatment group. Embryo gene expression analyses were performed using RNA extracted from 12 separate embryos, six of which were non-malformed and six of which exhibited malformations, from each experimental treatment group. RNA was extracted using Trizol Reagent (Invitrogen, Waltham, MA, USA). The samples were processed using an RNeasy Mini Kit (Qiagen, Hilden, Germany). The concentration and purity of the extracted RNA were determined by measuring the absorbance at 260 nm and 280 nm using a Nanovue spectrophotometer (GE healthcare, Chicago, IL, USA). The integrity of the RNA was assessed by agarose gel electrophoresis. Reverse transcription was performed using 500 ng of RNA with iScript cDNA synthesis kit (Bio-Rad Laboratories, Hercules, CA, USA) using random hexamer primers.

qPCR analyses were performed in a CXF96 (Bio-Rad Laboratories, Hercules, CA, USA) thermal cycler. cDNA obtained from 6.25 ng of reverse transcribed RNA were run in duplicate using 500 nM intron-skipping primers (sequences are listed in the Supplementary Table S2), and to tata-box binding protein (Tbp) as a housekeeping gene, and SYBR Green Master Mix (Takara Bio, Shiga, Japan). The expression of the non-malformed embryos from control mothers was set as 1 and relative expression was calculated using the Pfaffl method [44].

### 2.12. Murine Embryonic Stem Cell (ESC) Culture

Mouse D3 ESC (ATCC, Manassas, VA, USA) were cultured and induced to form neuronal precursors, resembling the embryonic neuroepithelium that forms the neural tube, as described [28]. Briefly, undifferentiated (UD) ESC was grown as monolayers, then were induced to form embryoid bodies, then differentiating (D) neuronal precursors were selected from embryoid bodies in attached cultures. D ESC cultures were terminated after four days cultured with or without 0.25 mmol/L buthionine sulfoximine (BSO), a gamma-glutamylcysteine inhibitor [45], to inhibit the synthesis of glutathione, or 0.001 µmol/L; antimycin A (AA), a mitochondrial complex III inhibitor to stimulate the production of superoxide (both from Sigma-Aldrich, St. Louis, MO, USA).

### 2.13. ESC Gene Expression Analysis

Total RNA was extracted using Trizol (Thermo Fisher, Waltham, MA, USA) and 200 ng was reverse transcribed, followed by real-time PCR using TaqMan^TM^ probes and primers for murine *Pax3* or *rRNA* as described [19], in a QuantStudio 3 instrument (Thermo Fisher, Waltham, MA, USA). *Pax3* mRNA was normalized to *rRNA* and expressed relative to expression by D cultures.

### 2.14. Statistical Analyses

Results are presented as mean ± SEM. For each variable, outliers were identified using the Rout test with a Q = 1%. Then, the normality of the distributions was assessed using the Kolmogorov–Smirnoff test. For Gaussian distributions, student’s *t* test or one-way analysis of variance (ANOVA), followed by post hoc Tukey’s multiple comparison tests, were applied. Otherwise, the Mann–Whitney test and Dunn’s multiple comparison test were respectively used. The statistical analysis was performed using GraphPad Prism (version 9, GraphPad Software, San Diego, CA, USA) and differences were considered statistically significant when *p* < 0.05.

## 3. Results

### 3.1. Vitamin E Supplementation Ameliorates Cafeteria Diet-Induced Insulin Resistance

**Figure 1:**
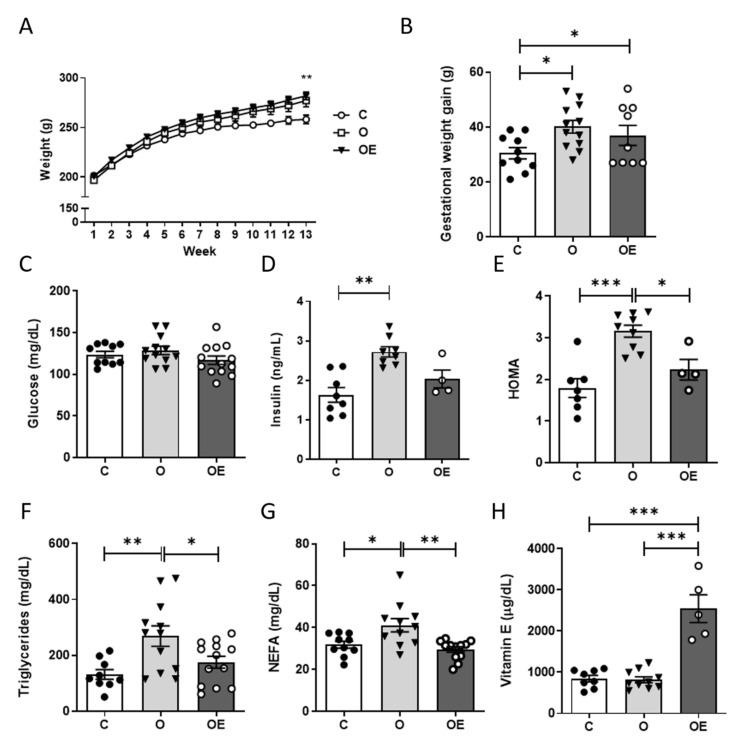
Vitamin E supplementation impedes metabolic dysregulation in high fat-fed pregnant rats. (**A**) Pregestational changes in body weight in response to an HF diet (29% energy from fat) during 13 weeks in non-supplemented animals (O; black triangles, *n* = 12) and supplemented with 150 mg of α-tocopherol twice a week by oral gavage (OE; white circles, *n* = 12) compared to controls fed on a standard diet (C; black circles, *n* = 10) before mating. (**B**) Weight gain between gestational day 0.5 and sacrifice on day 11.5. Fasting plasma concentrations of glucose (**C**) and insulin (**D**) measured in rats at sacrifice. (**E**) Homeostasis Model Assessment (HOMA), used as an indicator of insulin resistance. (**F**) Fasting plasma triglycerides and (**G**) non-esterified fatty acids (NEFA). (**H**) Plasma levels of α-tocopherol. Results are represented as mean + SEM. * *p* < 0.05; ** *p* < 0.01 *** *p* < 0.001.

Pregestational obesity was induced by feeding female Wistar rats with a cafeteria diet for 13 weeks. In that time, the differences in the weight of the O and OE rats became statistically significantly greater than the lean, C rats, which were fed with a standard diet (Figure 1A). Vitamin E supplementation did not have any effect on weight gain, as no differences were observed between the O and OE groups. Once pregestational obesity was reached, rats were mated and their weights were recorded during gestation. During the 11.5 days of gestation, there was significantly greater weight gain of the O and OE groups compared to the control group (Figure 1B).

Maternal metabolic parameters at the time when rats were sacrificed on day 11.5 of pregnancy were determined. No differences were observed in glucose concentrations among any of the three groups (Figure 1C). However, there was a significant increase in the circulating concentrations of insulin (Figure 1D) in the O group that was partially reversed by the administration of vitamin E. The HOMA index indicated insulin resistance in the O dams, which was ameliorated by vitamin E supplementation (Figure 1E). There was an increase in plasma triglycerides (Figure 1F) and free fatty acids (Figure 1G) in the O group compared with the C group, which was also prevented by vitamin E supplementation. We also found a three-fold increase in the circulating levels of vitamin E in the OE group in comparison to the other two groups (Figure 1H).

### 3.2. Vitamin E Supplementation Reduced Embryo Malformations Caused by Pregestational Obesity

**Figure 2:**
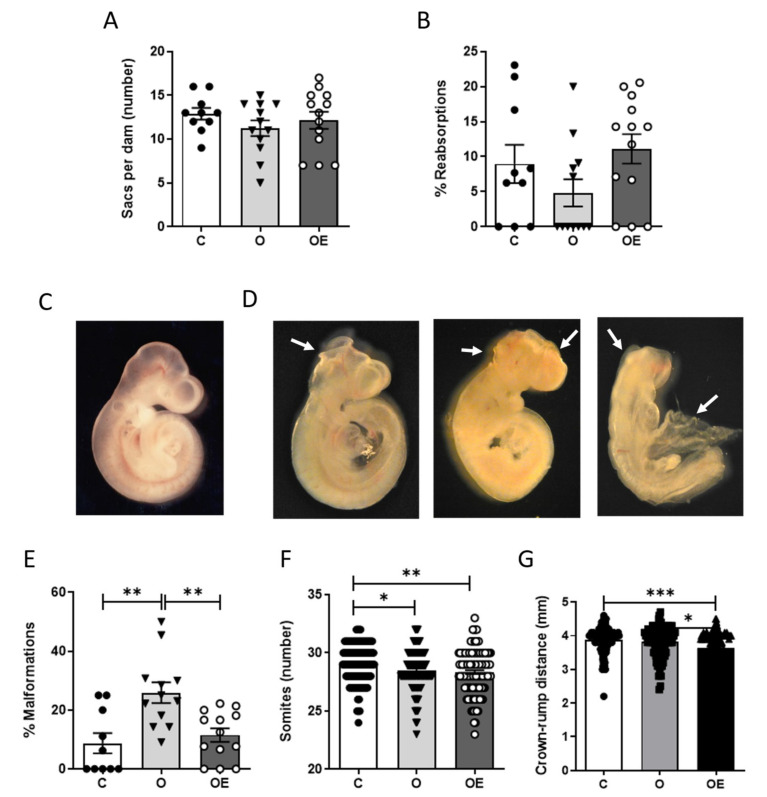
Vitamin E supplementation prevents obesity–induced malformations. After 13 weeks of dietary treatment, rats were mated. During gestation, corresponding diets were maintained, and vitamin E supplementation was administered daily. Embryos were dissected on day 11.5 of gestation. (**A**) Decidual sacs per dam. (**B**) Rate of resorptions. (**C**) Image of an embryo showing a normal morphology, with proper proportions of cerebral vesicles, complete neural tube closure, complete otic and cardiac vesicles and full axial curvature. (**D**) Embryos showing major malformations such as open neural tube (left), lack of cerebral vesicles (middle) or general agenesis of structures (right). (**E**) Rate of major malformations. (**F**) Numbers of somites/embryo (**G**) Crown-rump distances. Results are represented as mean + SEM.* *p* < 0.05; ** *p* < 0.01 *** *p* < 0.001. C: Black circles; O: Black triangles; OE: white circles.

Uteri were examined for numbers of implantation sites at sacrifice on day 11.5 of gestation. There were no differences in the numbers of sacs per dam among any of the three groups (Figure 2A), indicating that fertility was not affected by the dietary treatment or the vitamin E administration. No statistically significant differences were observed in the percentage of resorptions between groups (Figure 2B).

We next performed morphological analyses of the embryos as described in the Materials and Methods section. An example of a developmentally appropriate, normal embryo is shown in Figure 2C. Any embryo failing to exhibit any of these characteristics was considered malformed, examples of which are shown in Figure 2D. Pregestational obesity caused an average malformation rate of 25.91 ± 3.53% (30/125), compared to 8.74 ± 3.41% (10/115) in embryos of control, lean dams (Figure 2E). Most of the malformations that were observed were neural tube defects, such as open anterior and/or posterior neuropore. Cardiac defects and lack of axial rotation were also commonly observed. Notably, vitamin E supplementation prior to and during pregnancy reduced the embryo malformation rate to control levels (11.51 ± 2.31% (15/140)).

Both the crown-rump distances and the numbers of somites were significantly reduced in the OE embryos, compared to the C embryos (Figure 2F,G), suggesting that vitamin E treatment in the obese pregnancies caused a slight, but statistically significant, developmental and growth delay.

### 3.3. Obesity Decreases Free Radical Scavenging Capacity of Maternal Livers Which Is Restored by Vitamin E Supplementation

**Figure 3:**
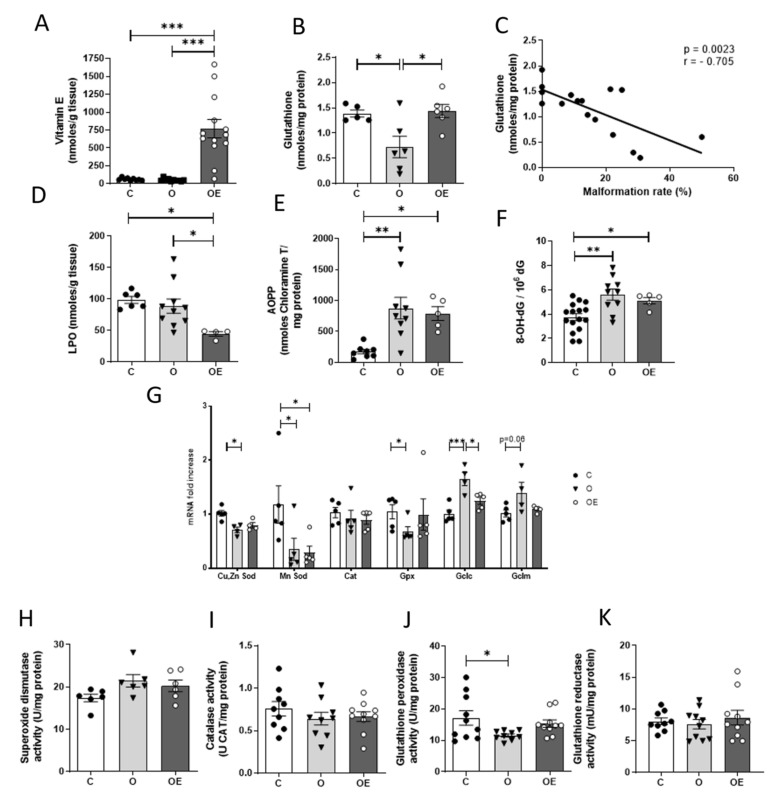
Cafeteria diet induces oxidative stress in the livers of obese, pregnant rats. Non-enzymatic antioxidants were determined in the livers of the pregnant rats: (**A**) vitamin E and (**B**) total glutathione. (**C**) Correlation between maternal liver total glutathione content and embryo malformation rate. Oxidative damage was analyzed through the quantification of oxidation end-products: (**D**) Hepatic lipoperoxides, (**E**) Advanced oxidation protein products (AOPPs), (**F**) 8-hydroxy-deoxyguanosine levels, as a marker of oxidative damage to DNA. (**G**) qPCR assays were performed of genes encoding free radical scavenging enzymes: *Cu, ZnSod, MnSod, Cat, Gpx, Gclm* and *Gclc*. Expression values represent 5 biological replicates and are shown relative to *Tbp* expression as a housekeeping gene. Activity assays of the antioxidant enzymes, superoxide dismutase (**H**), catalase (**I**), glutathione peroxidase (**J**) and glutathione reductase (**K**). Results are represented as mean + SEM. * *p* < 0.05; ** *p* < 0.01 *** *p* < 0.001. C: Black circles; O: Black triangles; OE: white circles.

Free radical scavenging and antioxidant capacities and markers of oxidative stress in maternal livers in the three treatment groups were next examined. The cafeteria diet did not induce any change in vitamin E content compared to the control, but there was an almost 10-fold increase in vitamin E content in the livers of OE rats compared to the other two groups (Figure 3A). Glutathione levels were significantly decreased in the O group, while vitamin E supplementation restored them to control levels (Figure 3B). Notably, there was a strong inverse correlation between the maternal hepatic glutathione levels and the rates of embryo malformation (Figure 3C).

To investigate whether the different dietary and vitamin E treatments had different effects on oxidation within the liver of the dams, two different approaches were taken. First, we measured the products of oxidative damage to tissue macromolecules, and then assayed expression and activities of free radical scavenging enzymes.

Lipid oxidation, determined by measurement of the two main end by-products, malondialdehyde and 4-hydroxyalkenals together, was decreased in the OE group compared to both the O and C groups (Figure 3D). However, markers of oxidative damage to proteins and to DNA were increased in the livers of the O and OE rats, and vitamin E supplementation did not prevent the oxidative damage to either proteins or DNA (Figure 3E,F).

The expression of genes encoding the free radical scavenging enzymes, Cu, Zn superoxide dismutase (Cu, ZnSod), Mn superoxide dismutase (MnSod), catalase (Cat), glutathione peroxidase (Gpx) and the catalytic and modifier subunits of the glutathione synthesizing enzyme, glutamate-cysteine ligase (Gclc and Gclm), were assayed by real time-PCR. There was decreased expression of *Cu, ZnSod*, *MnSod* and *Gpx* mRNA and increased expression of *Gclc* in the livers of O dams. The expression of mRNA of both *Sod* isoforms and *Gpx* in the livers of OE dams was decreased, but the expression of *Gclc* mRNA was comparable to that of control rats (Figure 3G). There were no differences in activities of catalase or superoxide dismutase enzymes among the three groups (Figure 3H,I). Glutathione peroxidase activity was decreased in the O group and was restored by vitamin E supplementation (Figure 3J). There were no differences in activities of glutathione reductase between the three groups (Figure 3K).

### 3.4. Glutathione Levels Are Decreased and Markers of Oxidative Stress Are Increased in Malformed Embryos from Obese Mothers

**Figure 4:**
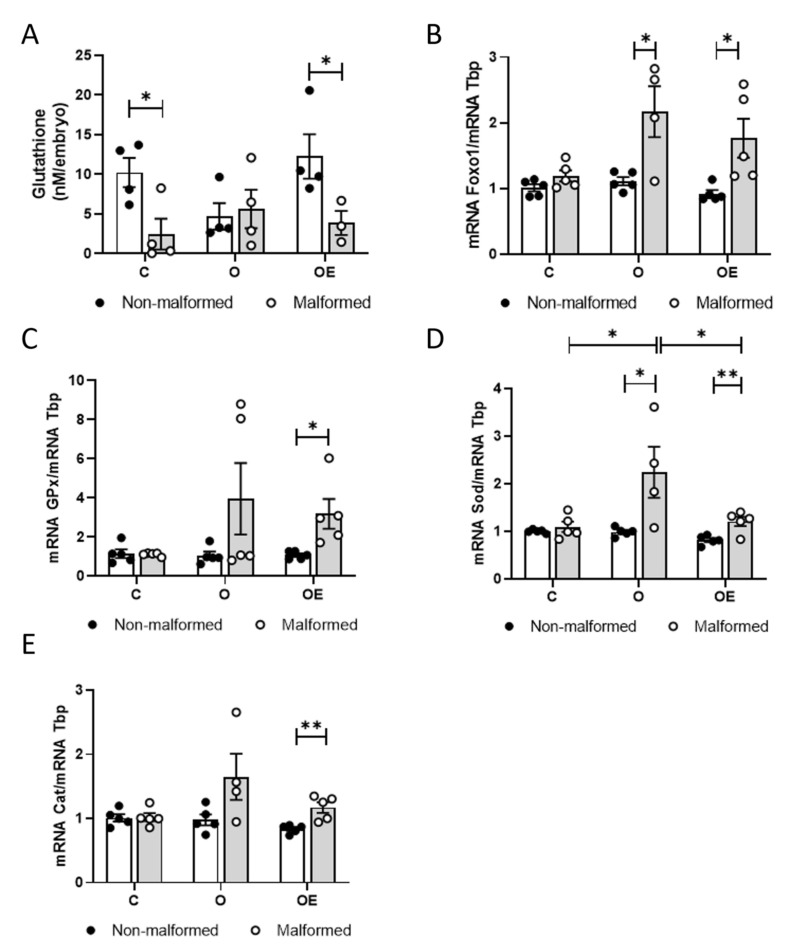
Oxidative stress is increased in malformed embryos of obese mothers. Twelve embryos of each group, 6 with (white circles) and 6 without malformations (black circles), were processed individually. (**A**) Glutathione content in the individual embryos. (**B**) qPCR assay of the gene encoding the transcription factor, Foxo1. mRNA levels encoding the antioxidant enzymes Gpx (**C**), Sod (**D**) and Cat (**E**). Expression values represent 6 biological replicates and are shown relative to *Tbp* expression as a housekeeping gene. Results are represented as mean + SEM. Student’s *t* test or Mann–Whitney test was used to compare between non-malformed and malformed embryos. One-way ANOVA followed by Tukey’s multiple comparison test or Dunn’s multiple comparison test was used to compare among treatments. * *p* < 0.05; ** *p* < 0.01.

Because OS can be teratogenic, we analyzed markers of OS in individual embryos, six malformed and six non-malformed, from each of the treatment groups. First, we investigated whether the decrease in maternal glutathione in the O dams could be associated with decreased embryo glutathione levels. We found that glutathione was decreased in the malformed embryos of C and OE mothers compared to the non-malformed embryos of these treatment groups. However, glutathione was decreased in both non-malformed and malformed embryos from the obese mothers (Figure 4A), suggesting decreased delivery of glutathione to embryos from obese mothers.

Then, we analyzed the embryo expression of the gene encoding the transcription factor, Foxo1, a gene whose expression is increased in response to OS [46,47], and genes encoding free radical scavenging enzymes. The mRNA levels of *Foxo1* remained unchanged in the embryos of C mothers, whether or not they were malformed. However, *Foxo1* mRNA was increased in the malformed embryos from both O and OE dams compared to non-malformed embryos (Figure 4B). *Gpx*, which is a target of Foxo1, displayed a similar pattern of expression as *Foxo1*, although the differences were only significant between the normal and malformed embryos of OE mothers (Figure 4C).

There were no differences in expression of the free radical scavenging enzyme encoding genes, *Sod* and *Cat*, between the normal and malformed embryos of C mothers. However, *Sod* expression was increased in the malformed embryos of O and OE mothers compared to non-malformed embryos, and *Cat* expression was only statistically increased in the malformed embryos of OE mothers compared to the non-malformed embryos. One-way ANOVA analyses in the malformed embryos from the three groups revealed an increase in *Sod* expression in the O group, suggesting an increased need for superoxide radical scavenging, which was modulated with the administration of vitamin E to the mothers. The same pattern was observed in the mRNA levels of *Cat*, although it was not statistically significant (Figure 4D,E).

### 3.5. Inhibiting Glutathione Synthesis Inhibits Pax3 Expression in a Cell Culture Model of Neuroepithelium

**Figure 5:**
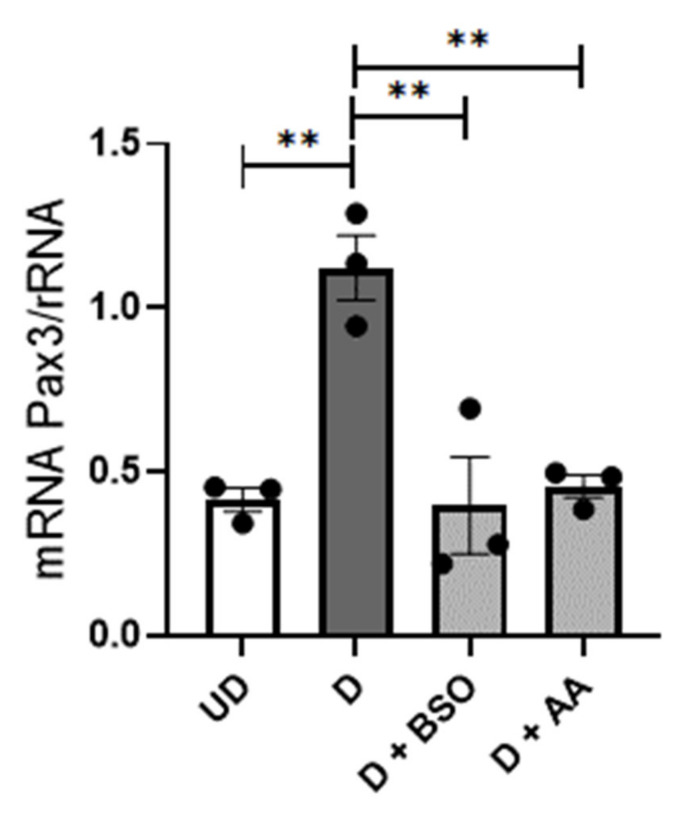
Inhibiting GSH synthesis in ESC-derived neuronal precursors inhibits *Pax3* expression. Real time reverse transcription-PCR of *Pax3* mRNA relative to *rRNA* from undifferentiated (UD) ESC and differentiating neuronal precursors (D) following four days of culture without or with 0.25 mM buthionine sulfoximine (BSO) or 0.001 µM antimycin A (AA). Data shown are the mean + SEM from triplicate culture dishes. ** *p* < 0.01.

Undifferentiated mESC (UD) are developmentally similar to the inner cell mass of the blastocyst, but they can be induced to form differentiating neuronal precursors (D) with developmental and gene expression profiles similar to those of neuroepithelium that gives rise to the neural tube [28]. We have previously shown that oxidative stress inhibits expression of *Pax3* in D mESC, as well as in embryos of diabetic mice, and that oxidative stress-mediated inhibition of *Pax3* leads to neural tube defects in embryos of diabetic mice [19,28]. To test whether there could be direct effects of glutathione depletion on embryo neuroepithelium leading to neural tube defects, mESC-derived D neuronal precursors were employed as a cell culture model. During the formation of neuronal precursors, cells were treated with buthionine sulfoximine (BSO) to inhibit the synthesis of reduced glutathione (GSH), and the expression of *Pax3* was assayed. As shown in Figure 5, *Pax3* expression was significantly increased in D ESC compared to UD ESC. *Pax3* induction was significantly inhibited by BSO, as well as by antimycin A, which was used as a positive control to induce OS. This suggests that GSH depletion induces oxidative stress, leading to impaired expression of genes that are needed for neural tube closure.

## 4. Discussion

In this study, we used a diet-induced obesity model in Wistar rats to investigate the role of pregestational obesity in teratogenesis and whether oxidative stress could be responsible for embryo malformation. To test this, we supplemented obese animals with the lipid–soluble antioxidant, vitamin E (α-tocopherol). We found that obesity significantly increased the rate of embryo malformation and that this appears to be due to decreased availability of glutathione to be transported from the mother to the embryo. Vitamin E supplementation of O dams inhibited the decrease in maternal glutathione, which decreased the rates of embryo malformations.

We have previously shown that the teratogenic effect of maternal diabetes can be prevented by vitamin E [18]. Because diabetes and obesity may share some characteristics, such as defective insulin signaling, and oxidative stress or inflammation, we aimed to understand the teratogenic effects of maternal obesity. To address this, we employed a Wistar rat model in which obesity is induced by feeding a HF diet for 13 weeks. Rats were then mated and sacrificed at day 11.5, corresponding to the end of the embryonic stage, to analyze embryo morphology. We found that 25.91% of embryos from obese mothers were malformed, while an 8.74% of malformation rate was found in the embryos of lean, control dams. This increase in malformations in the embryos of obese dams are consistent with the increased incidence of spina bifida, omphalocele, heart defects and other malformations observed in human pregnancies from overweight and obese mothers [48]. A recent report of more than 1 million liveborn infants demonstrated a direct relation between maternal body mass index (BMI) and rates of malformations, including heart, limb, digestive and nervous system defects [49]. An extension of this population-based cohort study published in 2019 revealed the relation between cardiac malformations and pregestational maternal obesity in more than 2 million newborns [50].

In our study, vitamin E supplementation of the obese dams reduced the malformations rate to 11.51%, with a resorption rate similar to that of control rats. However, vitamin E supplementation slightly (less than 5%) delayed embryo growth and development according to the somite number and crown-rump distance. This is a comparable reduction in the malformation rate that we achieved in diabetic rats using vitamin E supplementation [18]. Vitamin E has also been successfully used to reduce teratogenesis induced by valproic acid in mice [51]. In humans, retrospective, survey-based studies, suggest a direct relation between decreased antioxidant consumption and increased odds of limb and neural tube defects in obese pregnant women [52,53].

The mechanisms by which the physiopathology of obesity cause malformations is not fully understood. Metabolic impairment, oxidative stress and inflammation arise as potential mechanisms linking obesity and teratogenesis. First, the effect of hyperglycemia in teratogenesis has been widely studied in models of diabetic pregnancy [25,28,54]. The underlying mechanisms may include AMP-activated protein kinase (AMPK) activation by oxidative stress, with a concomitant inhibition of expression of *Pax3* [28], a key gene involved in neural tube and cardiac outflow tract development. *Pax3* is expressed in embryonic neuroepithelium and neural crest cells and embryos that are homozygous for *Pax3* loss-of-function alleles develop neural tube and cardiac outflow tract defects with 100% penetrance. Previous studies have shown that *Pax3* expression is significantly reduced in the embryos of diabetic mice through the induction of oxidative stress, and that administration of glutathione ethyl ester (GSH-EE) or vitamin E prevent the inhibition of Pax3 expression and increased neural tube defects [19]. This suggests that oxidative stress induced by maternal hyperglycemia phenocopies the effect of *Pax3* loss-of-function mutation. *Pax3* expression is induced in neuronal precursors formed from mESC, and expression is inhibited by inducing oxidative stress with the mitochondrial complex III inhibitor, antimycin A [28]. However, the normoglycemia present in our obese rats excludes glucotoxicity as a potential mechanism in obesity-induced teratogenesis. This is consistent with several reports that malformations are increased in the offspring of obese women even without a diagnosis of diabetes or with poor glycemic control [55,56].

Other metabolic disturbances, such as hypertriglyceridemia and elevated free fatty acids, that are present in our obese rats, have been proposed to cause embryo malformations [57], although there is a need for further research to provide stronger evidence and to determine mechanisms. Our obese rats were also hyperinsulinemic; however, it is thought that maternal hyperinsulinemia does not have a direct effect on embryo development [58,59]. On the other hand, hyperinsulinemia may increase ovarian progesterone synthesis, which can alter RNA storage and turnover in the oocyte [60], which may have an impact on the later embryogenesis.

Oxidative stress, defined as an imbalance between oxidants and antioxidants in favor of the oxidants, leading to a disruption of redox signaling and control and/or molecular damage [61], has been proposed to mediate the effects of several teratogens in addition to maternal diabetes [62,63,64]. During organogenesis, when embryos are susceptible to teratogens, glutathione may play a key role in maintaining the redox balance and preventing oxidative-induced embryopathy [65,66,67]. In our model, maternal glutathione was decreased in obese mothers, even despite the activation of a compensatory mechanism through the upregulation of the catalytic subunit of the glutamate–cysteine ligase, the key subunit in reduced glutathione synthesis. Both the activity and mRNA levels of the main glutathione oxidizing enzyme, GPx, were also diminished in the obese mothers. As a result, there was decreased availability of glutathione for the embryos, causing a higher rate of malformations and a reduced content of glutathione, even in the non-malformed embryos. It is important to note that all the malformed embryos, independently of the nutritional status of the mothers, contained less glutathione in comparison to normal embryos in the same treatment groups. The upregulation of the genes encoding free radical scavenging enzymes in the malformed embryos supports OS as a pivotal mechanism in obesity-induced teratogenesis. The supplementation with vitamin E successfully prevented the malformations, potentially by protecting from maternal GSH depletion, as has been previously described [22,68,69]. Inhibiting glutathione synthesis in a cell culture model of differentiating neuroepithelium inhibited *Pax3* expression, which is required to prevent p53-mediated apoptosis during differentiation of neuroepithelium and neural crest [25,26,27,29]. An effect of maternal obesity of inducing malformations by ferroptosis cannot be excluded, because p53 can induce cell death through ferroptosis, and vitamin E can inhibit ferroptosis induced by lipid oxidation [70,71,72]. However, because oxidative stress works upstream of *Pax3* expression through transcriptional regulation by AMPK and DNA methylation [73], maternal obesity-induced OS and inhibition of expression of essential genes in the embryo can provide a molecular explanation for the teratogenesis of maternal obesity.

Finally, inflammation has been suggested to play a key role in pregnancy loss, both early and recurrent, or modifying fetal brain development [74,75]. Inflammation has also been suggested as a plausible mechanism in teratogenesis, since high maternal and embryo levels of cytokines have been associated with embryo malformations [76,77]. In our model, maternal circulating and hepatic levels of the main proinflammatory cytokines remained unaltered (Supplementary Table S3), suggesting that the obesity-related teratogenesis of our high-fat diet model does not depend on inflammation.

## 5. Conclusions

In summary, our data show that pregestational obesity is teratogenic. The reduction in the malformation rate by vitamin E supplementation suggests that oxidative stress, mainly through maternal glutathione depletion and the resulting decreased availability for the embryo, is responsible. However, future investigations should provide a deeper insight from a mechanistic point of view. We believe that our model mimics the metabolic disturbances that occur in overweight or moderately obese human mothers, hence representing a suitable model for investigating obesity-induced teratogenesis and for testing treatments with potential anti-teratogenic effects.

## Data Availability

Data is contained within the article and supplementary material.

## Supplementary Materials

The following are available online at https://www.mdpi.com/article/10.3390/antiox10081173/s1, Table S1: Diet composition, Table S2: List of primers used in the qPCR studies, Table S3: Levels of circulating and hepatic cytokines.
