# Supplementary material for: Prevention of Teratogenesis in Pregnancies of Obese Rats by Vitamin E Supplementation
Source: Antioxidants (Basel). 2021 Jul 23;10(8):1173. doi: 10.3390/antiox10081173 (PMC8389020; doi:10.3390/antiox10081173)
Supplement: Supplementary file 1 [file antioxidants-10-01173-s001.zip › antioxidants-1259010-supplementary.pdf]

Supplementary Table S1. Diet composition.

Cafeteria diet composition

Ingredient	Content (%)
Ground commercial rat chow	33
Full fat condensed milk	35
White sugar	7
Muffins	10
Powdered milk	15

Caloric content

	Standard chow diet (C group)	Cafeteria diet (O and OE groups)
Kcal/100g	194.3	242.1
% Energy from carbohydrates	67	58.6
Kcal/100g	37.7	118.6
% Energy from fat	13	28.7
Kcal/100g	58	52.4
% Energy from protein	20	12.7
Kcal/100g	290	413.1
Total (%)	100	100

Supplementary Table S2. List of primers used in the qPCR analysis.

Gene	Accession number	Forward (5´-3´)	Reverse (5´-3´)
Cat	NM_012520.2	TCAGGGATGCCATGTTGTT	GGGTCCTTCAGGTGAGTTTG
Cu, Zn Sod	NM_017050.1	GGTCCAGCGGATGAAGAG	GGACACATTGGCCACACC
Foxo1	NM_001191846.2	TCAGGCTAGGAGTTAGTGAGCA	GGGGTGAAGGGCATCTTT
Gclc	NM_012815.2	TTGTCGCTGGGGAGTGATTT	TGATTTTAAAGCGATTGTTCTTCAG
Gclm	NM_017305.2	TGACTCACAATGACCCAAAAGA	CCTCTGCTTTTCACGATGAC
Gpx	NM_022525.4	ATTCTGGGCTTCCCTTGC	CACCCGGTCGAACGTACT
Mn Sod	NM_017051.2	CACCGAGGAGAAGTACCACGA	TGGCCCCCGCCATTGAACTT
Tbp	NM_001004198.1	CCCACCAGCAGTTCAGTAGC	CAATTCTGGGTTTGATCATTCTG

Supplementary Table S3. Levels of circulating and hepatic cytokines.				
Plasma				
		C	O	OE
	MCP-1 (pg/ml)	181.8 ± 45.83	230.90 ± 19.58	169.8 ± 14.28
	TNF-α (pg/ml)	2.51 ± 0.49	2.90 ± 0.31	1.81 ± 0.17
	IL-6 (pg/ml)	61.16 ± 28.53	81.36 ± 17.47	58.45 ± 17.51
	PAI-1 (pg/ml)	22.16 ± 3.77	16.36 ± 1.72	12.18 ± 3.32
Liver				
		C	O	OE
	MCP-1 (pg/g protein)	11.39 ± 3.39	22.50 ± 7.90	15.96 ± 5.34
	TNF-α (pg/g protein)	3.61 ± 0.52	5.44 ± 0.76	7.52 ± 2.95
	IL-6 (pg/g protein)	116.50 ± 8.20	185.20 ± 32.07	104.5 ± 23.10
	PAI-1 (pg/g protein)	1.88 ± 0.76	3.09 ± 1.57	4.88 ± 3.86
